# Antihypertensive Deprescribing in Older Adults: a Practical Guide

**DOI:** 10.1007/s11906-022-01215-3

**Published:** 2022-07-26

**Authors:** James P. Sheppard, Athanase Benetos, Richard J. McManus

**Affiliations:** 1 Nuffield Department of Primary Care Health Sciences, University of Oxford, Radcliffe Primary Care Building, Radcliffe Observatory Quarter, Oxford, OX2 6GG UK; 2 Maladies du Vieillissement, Gérontologie Et Soins Palliatifs”, and Inserm DCAC u1116, CHRU-Nancy, Université de Lorraine, 54000 PôleNancy, France

**Keywords:** Blood pressure, Drug-related side effects and adverse reactions, Polypharmacy, Ageing, Medication review, Evidence-based medicine

## Abstract

**Purpose of Review:**

To summarise evidence on both appropriate and inappropriate antihypertensive drug withdrawal.

**Recent Findings:**

Deprescribing should be attempted in the following steps: (1) identify patients with several comorbidities and significant functional decline, i.e. people at higher risk for negative outcomes related to polypharmacy and lower blood pressure; (2) check blood pressure; (3) identify candidate drugs for deprescribing; (4) withdraw medications at 4-week intervals; (5) monitor blood pressure and check for adverse events. Although evidence is accumulating regarding short-term outcomes of antihypertensive deprescribing, long-term effects remain unclear.

**Summary:**

The limited evidence for antihypertensive deprescribing means that it should not be routinely attempted, unless in response to specific adverse events or following discussions between physicians and patients about the uncertain benefits and harms of the treatment.

**Perspectives:**

Clinical controlled trials are needed to examine the long-term effects of deprescribing in older subjects, especially in those with comorbidities, and significant functional decline.

## Introduction

High blood pressure (hypertension) is the leading modifiable risk factor for cardiovascular disease and the most common condition in older people with multiple long-term conditions [1]. Hypertension can be effectively treated with antihypertensive medication which has been shown to reduce the risk of stroke and cardiovascular disease across age groups [2]. As a result, treatment is commonly prescribed [3], particularly in older patients, where more than half of individuals aged 80 years or older are treated with antihypertensive therapy [4].

However, antihypertensive treatment is not without harm. A recent meta-analysis of 58 randomised controlled trials showed that treatment is associated with an increased risk of hypotension, syncope, acute kidney injury and hyperkalaemia [5]. Such adverse events are typically more common in older people, in part because they are more sensitive to the effects of medications due to altered pharmacokinetic and pharmacodynamic responses [6]. They are also more likely to be prescribed multiple medications leading to polypharmacy [7], which increases the risk of drug-drug interactions. As a result, clinical guidelines for the management of hypertension recommend using clinical judgement when prescribing in frail older people, emphasizing a personalised approach to care [8–10]. Indeed, for some older patients, it may become less important to maximise longevity and more important to prioritise other patient-centred outcomes with the goal of remaining functionally independent.

One proposed approach to achieve these patient-centred goals is through reducing the number of medications an individual is prescribed, known as deprescribing [11]. Because antihypertensives are one of the most commonly prescribed medications in older people [3], they are frequently proposed as a target for deprescribing. The concept of deprescribing is relatively new [11], and research on antihypertensive deprescribing is evolving quickly [12]. The present review will summarise the current evidence for the practice of antihypertensive deprescribing and make practical recommendations as to how this should be approached in routine clinical practice.

## What Is Deprescribing?

Deprescribing is described as the process of withdrawing an inappropriate medication, supervised by a healthcare professional with the goal of managing polypharmacy and improving outcomes [11]. Important elements of deprescribing which distinguish it from non-adherence or simply stopping potentially effective treatment are that it is “supervised” by a healthcare professional and it targets inappropriate medication [11]. In the context of the management of hypertension, an inappropriate antihypertensive medication would be one where the risks of an adverse event with treatment (e.g. falls, syncope and acute kidney injury) [5] outweigh the potential benefits (prevention of a stroke or myocardial infarction) [2]. Here, deprescribing might be attempted for the benefit of the patient, to prevent an adverse event from occurring in the future.

An antihypertensive medication might also be inappropriate if continued prescription does not align with the goals of care, for example in patients at the end of life, where the likelihood of accruing any further benefit (in terms of cardiovascular disease prevention) from treatment is very small [13]. Here, deprescribing might be attempted in the face of therapeutic futility.

In both situations, physicians must have an understanding of the benefits and harms of antihypertensive treatment, the timescales of such effects, and their impact in the specific population of interest.

## Understanding the Benefits and Harms of Antihypertensive Treatment

The best evidence for the benefits and harms of antihypertensive treatment comes from randomised controlled trials, which, combined, include hundreds of thousands of people [2, 5]. These show that treatment is associated with benefit in terms of reduced risk of stroke, myocardial infarction, cardiovascular disease and all-cause mortality [2, 14] and also a small risk of harm [5]. However, such trials are typically undertaken in younger populations, less likely to experience adverse events [15]. Even trials of antihypertensive therapy in older people tend to be targeted at healthier populations, less likely to have frailty, multi-morbidity and polypharmacy, excluding those with limited life expectancy [16]. Indeed, a recent analysis comparing participants in trials of antihypertensive therapy to similar patients residing in the community, found much lower rates of serious adverse events reported in trials, suggesting that these patients were fitter and healthier than the general population [17]. As a result, physicians have very little information about the benefits and harms of treatment in older people with multi-morbidity and frailty. This creates a situation of clinical inertia, where medications are sustained so as to continue the status quo [18]. Due to the lack of evidence for the efficacy of medication continuation in such populations, it is unclear whether this clinical inertia is appropriate or not.

## When Should Deprescribing Be Considered?

The most common indication that an antihypertensive medication may be inappropriate is following an adverse event thought to be caused by that medication. This is typically a reason for deprescribing antihypertensives in nursing homes [19] and may be considered if someone is admitted to hospital with a serious fall, syncope, acute kidney injury or electrolyte abnormalities such as hyperkalaemia (with concurrent prescription of medications affecting the renin-angiotensin system) or hypokalaemia (with concurrent prescription of a thiazide and thiazide-like diuretic). However, in many ways, deprescribing following an adverse event is too late.

Therefore, a priority of deprescribing is to identify inappropriate medications to withdraw in high risk patients before they lead to an adverse event. This is not straightforward, since it is difficult to know who is at high risk and such an approach is not without harm, since withdrawing an antihypertensive to prevent a fall could precipitate a “worse” cardiovascular event such as a stroke. These issues were considered in a recent review by Scott et al. [12], which suggested that antihypertensive deprescribing may be considered in patients over the age of 80 years but with no history of cardiovascular disease, moderate to severe frailty or cognitive impairment, a high risk of risk of syncope or falls, or those with life-limiting illness (e.g. end-stage diseases and metastatic cancer). Once again, this raises the question of how one should define moderate to severe frailty, cognitive impairment or indeed how to identify those with a high risk of falls/syncope. Similarly, one might need to consider how long a person would need to live in order for the balance of probability to be in favour of continued preventative treatment. While there are now tools for measuring frailty using information routinely available in electronic health records [20], further research is needed to develop robust methods for identifying candidates for antihypertensive deprescribing.

Another situation in which to consider antihypertensive deprescribing is in older people with low systolic blood pressure. While individuals with long-term lower blood pressure, not due to a co-morbidity, are likely to be at lower risk of cardiovascular disease [21], others can become symptomatic and/or develop orthostatic hypotension. These are associated with syncope and serious falls resulting in hospitalisation and even death. The PARTAGE study of older people residing in nursing homes found low blood pressure (< 130/69 mm Hg) to be associated with an increased risk of mortality in the next 2 years, [22] particularly in those prescribed two or more antihypertensive medications [23]. Similarly, people admitted to hospital with syncope or orthostatic hypotension were at increased risk of all-cause mortality and cardiovascular events (syncope) and stroke (orthostatic hypotension), highlighting the difficult balance between prevention of both cardiovascular disease and adverse events [24].

Furthermore, a recent systematic review of observational studies found that normal blood pressure (< 140/90 mm Hg) is associated with a reduced risk of mortality in the general older population without frailty, but not in those with frailty [25]. For these patients, it has been suggested that treatment may be prescribed to a higher blood pressure target [10], and this may require deprescribing in those already receiving antihypertensive medication [26, 27]. However, older patients with higher blood pressure are also at higher risk of cardiovascular disease, and therefore more likely to benefit from continued treatment [28]. Therefore, deprescribing should not be attempted in patients with uncontrolled blood pressure, which is typically defined as greater than 150/90 mm Hg for older patients aged 80 years and above. [9, 29]

## How Should We Deprescribe?

**Fig. 1:**
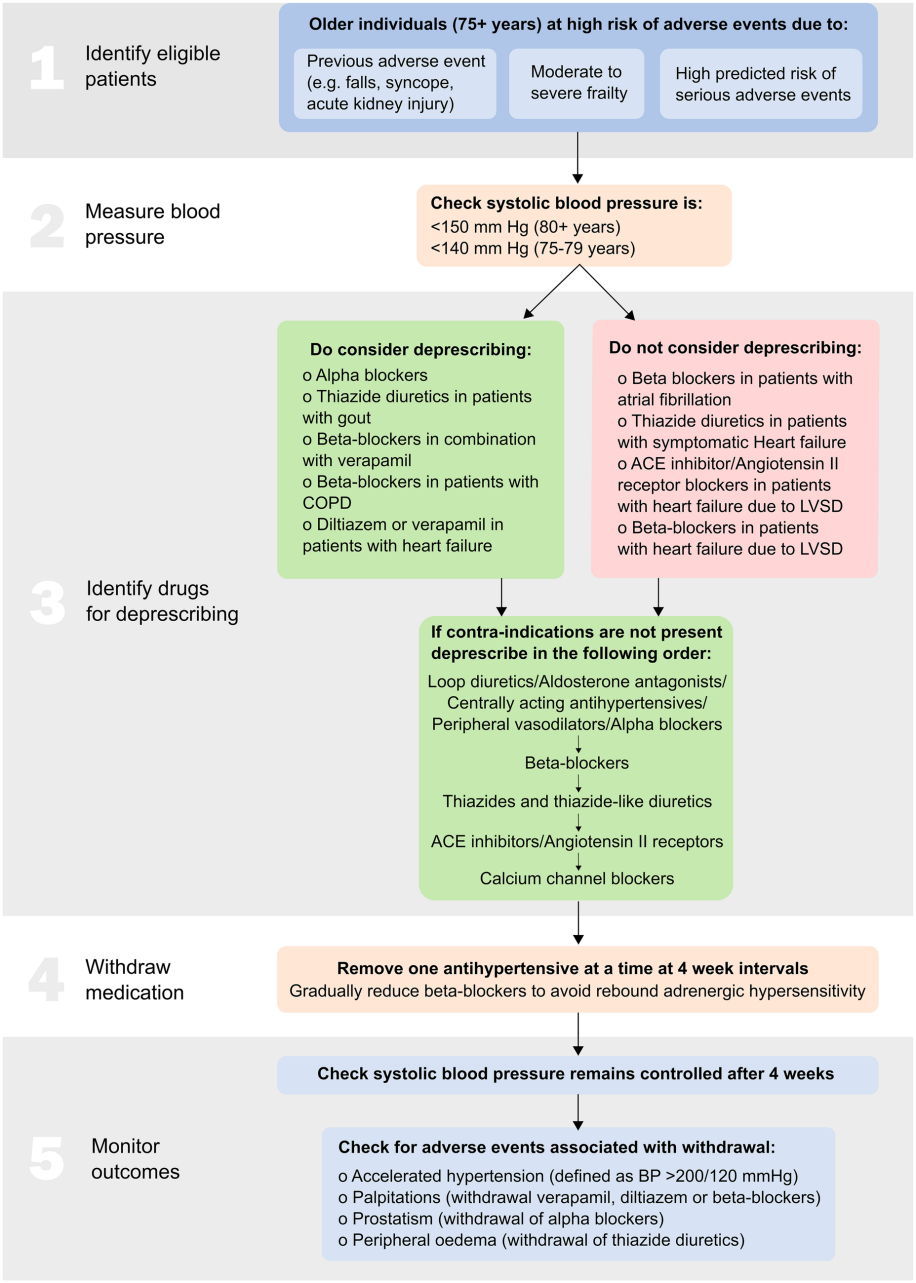
Antihypertensive deprescribing algorithm. ACE, angiotensin-converting enzyme; LVSD, left ventricular systolic dysfunction; COPD, chronic obstructive pulmonary disease; BP, blood pressure

Ideally deprescribing should be undertaken by a qualified pharmacist or physician with experience managing hypertension in older people. The process should involve 5 steps, focusing on the characteristics of the individual and careful monitoring of blood pressure and adverse events. These steps are summarised below and in Fig. 1.

1. Identify eligible patients
  As described in the previous section, patients are likely to be eligible for antihypertensive deprescribing if they are older (e.g. aged 75 + years) and at high risk of adverse events. Patients may be at high risk due to a previous adverse event in the preceding 12 months, or because they have developed moderate to severe frailty, functional decline and loss of autonomy. Such frailty may be identified using frailty measures such as the electronic frailty index or the clinical frailty scale [20, 30]. Healthcare professionals may also wish to use risk prediction tools to identify those with a high risk of specific adverse events such as falls. Although a number of tools exist [31], at present, very few have been externally validated and currently it is unclear what level of falls risk is sufficient to warrant antihypertensive deprescribing. Having identified potentially suitable patients, before proceeding further, clinicians should discuss the potential pros and cons with patients and consider a shared decision making approach [32].
2. Measure blood pressure
  In patients potentially eligible for deprescribing, blood pressure should be measured to check that it is controlled below guideline recommended levels before withdrawing treatment. Typically, this should be a clinic systolic blood pressure below 150 mm Hg (80 + years) or 140 mm Hg (75–79 years) [9, 10, 29]. Deprescribing is more likely to be tolerated in patients with a lower systolic blood pressure, such as readings below 130 mm Hg. In cases of life limiting illness, the threshold for intervention may be different due to the futility of treatment at all but the highest levels of blood pressure.
3. Identify candidate drugs for deprescribing
  To identify candidate drugs for deprescribing, an individual’s currently prescribed medications should be reviewed to identify antihypertensives which may have become contraindicated due to concomitant prescriptions or newly developed conditions. Such contraindications can be identified using published tools such as the STOPP/START [33] and STOPPFrail2 [34] criteria. In particular, these recommend stopping alpha blockers and other centrally acting vasodilators due to the risk of vasodilatation resulting in postural hypotension and falls [34]. Other contraindicated medications could include thiazide and thiazide-like diuretics in patients with a history of gout (may exacerbate gout), beta-blockers in combination with verapamil (risk of symptomatic heart block), non-cardioselective beta-blockers in patients with chronic obstructive pulmonary disease (risk of bronchospasm), calcium channel blockers in patients with chronic constipation (may exacerbate constipation) and diltiazem or verapamil in patients with advanced heart failure (may worsen heart failure) [33].
  In several cases, it may be inappropriate to deprescribe antihypertensive medications, particularly if they have been prescribed for indications other than to lower blood pressure. Examples could include beta-blockers prescribed in patients with atrial fibrillation, diuretics (loop diuretics, thiazide and thiazide-like diuretics) in patients with symptomatic heart failure and ACE inhibitors/angiotensin II receptor blockers/aldosterone antagonists or beta-blockers in patients with heart failure with low left ventricular ejection fraction.
  In the absence of any potentially contraindicated prescriptions, healthcare professionals should identify all prescribed antihypertensive medications and withdraw them one by one in *reverse* of guideline recommended treatment [35]. Typically this would result in first withdrawing medication that generally are not recommended in older adults such as loop diuretics, aldosterone antagonists, centrally acting antihypertensives, peripheral vasodilators and peripheral alpha blockers. Among the most commonly used drugs, beta-blockers could be the first to stop, followed by thiazide and thiazide-like diuretics, ACE inhibitor/Angiotensin II receptor blockers (2^nd^ line therapy for hypertension in older people) and finally calcium channel blockers (1^st^ line therapy for hypertension in older people) [9]. Such an approach is supported by a recent secondary analysis of a randomised controlled trial [35, 36], which showed that withdrawal of beta-blockers was associated with no change in systolic blood pressure, whereas withdrawal of calcium channel blockers was associated with an increased risk of uncontrolled blood pressure at 12-week follow-up [36]. This same analysis also suggested that removal of low dose medications may be associated with smaller increases in blood pressure at follow-up [36].
4. Withdraw medication
  Antihypertensives should be withdrawn in order of preference for deprescribing, one at a time at 4-week intervals. If withdrawing beta-blockers, healthcare professionals should consider first reducing the dose, before removing the drug altogether to avoid rebound adrenergic hypersensitivity. A similar progressive strategy could be also applied when withdrawing diuretics, especially in patients prescribed high doses of loop diuretics (e.g. > 40 mg of Furosemide) in order to avoid harms related to salt/water retention, and any other antihypertensive medications prescribed at high doses (e.g. amlodipine 20 mg, enalapril 40 mg and irbesartan 300 mg). It is important to check the patient’s systolic blood pressure 4 weeks after withdrawing therapy, to ensure it remains below target [9, 10, 29]. If it has become uncontrolled, healthcare professionals should consider re-introducing the medication previously withdrawn at a lower dose (if available), or recommend other non-pharmacological approaches to reduce blood pressure [37].
5. Monitor outcomes
In addition to monitoring blood pressure 4 weeks after deprescribing, healthcare professionals should check for adverse events associated with drug withdrawal. These might include signs of accelerated hypertension (defined as a blood pressure > 180/110 mm Hg), palpitations (following withdrawal of heart rate-limiting drugs such as verapamil, diltiazem or beta-blockers), prostatism (following withdrawal of alpha blockers) and peripheral oedema (following withdrawal of loop diuretics, thiazide and thiazide-like diuretics). Once again, if such signs and symptoms are present, healthcare professionals should consider re-introducing the medication previously withdrawn at a lower dose. If symptoms persist, it is important to seek expert advice.

## What Are the Potential Benefits and Harms of Deprescribing?

The evidence supporting antihypertensive deprescribing was recently examined in a Cochrane systematic review of randomised controlled trials [38]. This identified six trials including 1073 participants, but due to the low number of outcome events, found no association between antihypertensive deprescribing and all-cause mortality (4 studies, 18 outcome events), myocardial infarction (2 studies, 3 events), stroke (3 studies, 5 events) and all-cause hospitalisation (1 study, 19 outcome events) [38]. Indeed, there have been very few trials of antihypertensive deprescribing and even those published in the past 5 years have included very little data on important clinical outcomes (Table 1).

**Table 1:** Recent randomised controlled trials of antihypertensive deprescribing published in the last 5 years

Author	Trial name	Age range	Sample size	Population	Follow-up period	Proportion attempting to deprescribe medications	Proportion maintaining deprescribed medications	Mean difference in change in SBP	Clinical outcomes reported	
									**Deprescribing group**	**Control group (usual care)**
Luymes et al. [39]	ECSTATIC	40–70 years	1067	Community dwelling	24 months	65%	27%	4.9 mm Hg	2 CVD events	8 CVD events
Gulla et al. [40]	COSMOS	≥ 65 years	295	Nursing home residents	9 months	23%	21%	n/a	36 deaths 14 hospital admissions	29 deaths 26 hospital admissions
Sheppard et al. [35, 38]	OPTiMISE	≥ 80 years	569	Community dwelling	3 months	100%	66%	3.4 mm Hg	2 deaths 12 hospital admissions	7 hospital admissions

*SBP* systolic blood pressure, *CVD* cardiovascular disease

Another limitation of recent trials is that many fail to achieve antihypertensive deprescribing in the study intervention groups. For example, the recent Evaluating Cessation of STatins and Antihypertensive Treatment In primary Care (ECSTATIC) trial [39] examined the effect of deprescribing cardiovascular medications in community dwelling patients aged 40–70 years, and while 65% of the 1067 participants did stop a statin or antihypertensive, only 27% were able to maintain this throughout 2-year follow-up. Owing to the low risk population, only 10 cardiovascular events were reported across both study groups throughout follow-up [39].

The COmmunication, Systematic assessment and treatment of pain, Medication review, Occupational therapy, Safety (COSMOS) study [40] was not designed as an antihypertensive deprescribing trial but did examine the effect of an educational intervention on antihypertensive use in 295 participants. This study only achieved antihypertensive deprescribing in 23% of participants, although most of these were able to sustain fewer medications throughout 9 month follow-up. No comparisons were made between groups with regard to blood pressure at follow-up, although hospitalisations were reported to be higher in the control group at 4 month (14 [control] vs 7 [intervention]) and 9 month (12 vs 7) follow-up [40].

The most recent trial of antihypertensive deprescribing was the OPtimising Treatment for MIld Systolic hypertension in the Elderly (OPTIMISE) trial [35], which examined the short-term safety and efficacy of antihypertensive deprescribing. This trial examined withdrawal of one antihypertensive, in patients aged ≥ 80 years, with systolic blood pressure < 150 mmHg at baseline and prescribed two or more antihypertensive treatments. In 569 participants, 100% of those randomised to the intervention group deprescribed therapy, and 66% of these maintained this medication reduction throughout 12-week follow-up. No difference was observed in the proportion of patients with controlled blood pressure at follow-up (RR 0.98, 95% CI 0.92 to ∞) and nor was there any difference in serious adverse events (leading to hospitalisation or death), although once again the number of events was low (7 [control] vs 12 [intervention]) [35].

These trials, like those included in the preceding Cochrane review [38], had follow-up periods which were short or included too few high risk patients, and so were not powered to detect differences in clinical outcomes such as cardiovascular events or death. These data therefore cannot be used to determine whether antihypertensive deprescribing should be attempted in older patients with frailty. In the absence of robust effect estimates from randomised controlled trials, it is sometimes helpful to examine evidence from well-conducted observational studies. However, in addition to the usual issues of confounding by indication, observational studies of deprescribing can be particularly challenging due to difficulties defining a baseline time point for each included patient and selecting an appropriate comparator group [41].

These issues are illustrated in the recent study by Aubert et al. [42], which showed that antihypertensive deprescribing may be associated with an increased risk of cardiovascular, syncope and fall events, compared to continued prescribing. In this study, deprescribing was defined as fewer antihypertensive prescriptions 90 days after the index date (defined as the date of the second consecutive clinic visit with systolic blood pressure < 130 mmHg and ≥ 1 antihypertensive medication prescribed within a 2-year period). However, this definition is prone to misspecification in electronic health record data, since prescriptions are often not issued in a uniform manner and so any temporary gaps in prescription may inappropriately be attributed as deprescribing. Indeed, the fact that deprescribing was associated with an increased risk of both falls and cardiovascular events [42] (opposing outcomes of deprescribing) suggests that these patients were generally sicker and that some unmeasured confounding may have been present in this analysis [24].

One small observational study [43], which did utilise an appropriate time point to define baseline, examined the association between deprescribing upon hospital discharge and 90 day mortality. This analysis found a borderline increased risk of mortality following deprescribing of antihypertensives (OR = 2.27, 95% CI: 1.004, 5; *p* = 0.049) in 48 patients, compared to 132 patients who did not deprescribe [43]. Most recently, a post hoc analysis of the Trial of Nonpharmacologic Interventions in the Elderly (TONE) trial [44] attempted to examine the association between antihypertensive deprescribing and adverse events in 975 patients followed for up to 3 years. This was a trial of different weight loss and salt reduction interventions following which antihypertensive therapy was stopped [44]. However, since treatment was deprescribed in all four study groups, the between group comparisons made in this analysis do not give any insight into the effect of deprescribing on outcomes.

The current state of knowledge clearly shows the need for more evidence of the long-term benefits and harms of deprescribing. It is important to assess these effects both in robust elderly subjects but also in individuals with multiple comorbidities, cognitive disorders, functional decline and loss of autonomy, that is, patients who have hitherto always been excluded from long-term interventional trials [16, 28, 45]. Such trials are ongoing (Table 2), the largest of which are the RETREAT-FRAIL [46] and OPTIMSE2 trials. RETREAT-FRAIL is evaluating the long-term effects of deprescribing antihypertensive therapy in nursing home residents over the age of 80 with low blood pressure (< 130 mm Hg) treated with at least 2 antihypertensive drugs. The trial hypothesises that a gradual reduction of antihypertensive treatment in these very frail patients can improve survival (primary endpoint) during a follow-up period of an average of 3 years, by a controlled increase in systolic blood pressure and a decrease in secondary morbidity due to overmedication. Patients included in this trial are randomised into one of two parallel arms: the intervention arm will involve a tapering of antihypertensive medication, while the control arm will include standard antihypertensive therapy. The results of this study will be communicated in 2024.

**Table 2:** Ongoing or planned trials of antihypertensive deprescribing

Trial name	Country	Study design	Age group	Sample size	Population	SBP eligibility criteria	Intervention	Primary outcome	Planned completion date
DANTON	Netherlands	RCT	Not specified	492	Nursing home residents with dementia	< 160 mmHg	Antihypertensive discontinuation	Neuropsychiatric symptoms/quality of life	2022
OPTiMISE-X	UK	RCT (passive follow-up)	≥ 80 years	569	Community dwelling	< 150 mmHg	Medication reduction (one drug only)	All-cause hospitalisation or death	2023
RETREAT-FRAIL	France	RCT	≥ 80 years	1100	Nursing home residents	< 130 mmHg	Step-down medication reduction	All-cause mortality	2024
OptimizeBP	Canada	RCT	≥ 70 years	383	Nursing home residents	< 135 mmHg	Step-down medication reduction	All-cause mortality	2024
OPTIMISE2	UK	RCT	≥ 75 years	3014	Community dwelling at high risk of adverse events	< 150 mmHg	Step-down medication reduction	All-cause Emergency hospitalisation	2027

*SBP* systolic blood pressure, *RCT* randomised controlled trial

The OPTIMISE2 trial begins in the summer of 2022 and will examine a similar intervention to that being studied in RETREAT-FRAIL, but in patients aged 75 + years, living in the community who are at high risk of adverse events. Participants will have a baseline systolic blood pressure of less than 150 mm Hg and be taking at least two antihypertensive medications. The primary outcome will be all-cause emergency hospitalisation and participants will be followed-up for at least 1 year to examine whether antihypertensive deprescribing is non-inferior to usual care (i.e. continued treatment).

## Is Deprescribing Cost-effective?

Given the lack of evidence for antihypertensive deprescribing from trials and observational studies, one alternative approach to understanding the long-term effects is to model them as part of a cost-effectiveness analysis. Such analyses are uncommon in deprescribing research, and until recently were limited to studies examining the cost-effectiveness of nonsteroidal anti-inflammatory drugs and sedatives [47, 48]. These found deprescribing to be a cost-effective intervention, both in terms of saving money and increasing health-related quality of life.

The first cost-effectiveness analysis of antihypertensive deprescribing was undertaken using data from the ECSTATIC trial [39]. In patients with a low risk of cardiovascular disease, aged 40–70 years, attempting to deprescribe antihypertensives and statins was no more expensive than usual care, but resulted in a small improvement in quality of life (0.016 quality adjusted life years [QALYs]) over the 2-year time horizon of the trial [39]. This meant that at a willingness-to-pay of £20,000 per QALY gained, deprescribing had a 70% probably of being the cost-effective strategy. However, in older patients with a high risk of cardiovascular disease, deprescribing is less likely to be cost-effective due to the impact a cardiovascular event can have on healthcare costs and quality of life. A recent cost-effectiveness analysis based on the OPTiMISE trial [35, 49] examined outcomes over a lifetime time horizon and found antihypertensive deprescribing to be cost saving in older adults (aged 80 + years), but resulted in fewer quality adjusted life years gained when compared to usual care [49]. Therefore, usual care was the cost-effective strategy at £2975 per QALY gained compared to deprescribing, driven by an increased cardiovascular risk in the deprescribing group. Sensitivity analyses suggested that the deprescribing strategy may be preferred when targeted at individuals at high risk of adverse events (e.g. serious falls, acute kidney injury), but a lack of robust data regarding the underlying risk in this population, and the long-term effects of deprescribing precluded reliable conclusions being drawn.

## Conclusions

The concept of deprescribing is still relatively new [11] and as such, many aspects of this practice require further research before it can be recommended in routine clinical practice. In the context of antihypertensive deprescribing, well-conducted randomised controlled trials are urgently needed to determine the long-term effects on important clinical outcomes and health-related quality of life. These trials should also examine some of the more nuanced outcomes of deprescribing, such as the effects on medication burden, patient independence and restricted activity [50]. Such trials are ongoing and planned (Table 2), but it may be some time until the results of these studies are published. In the meantime, well-conducted observational studies [41] would provide physicians with useful information about the benefits and harms of deprescribing.

The present review has summarised the current evidence on the benefits and harms of antihypertensive deprescribing. While many of these remain unknown, studies have now shown that deprescribing is possible in both community and nursing home settings, and appears to be safe in the short term. These studies provide a framework for deprescribing, including how to identify eligible patients and potentially inappropriate medications, as well as how to withdraw treatment and monitor outcomes. However, the current lack of evidence on the long-term effects of antihypertensive deprescribing means that such an approach should not be routinely attempted, unless in response to specific adverse events or following informed discussions between physicians and patients about the uncertain benefits and harms of such an approach [32].
